# Supplementary material for: Compared Inhibitory Activities of Tamoxifen and Avenanthramide B on Liver Esterase and Correlation Based on the Superimposed Structure Between Porcine and Human Liver Esterase
Source: Int J Mol Sci. 2024 Dec 11;25(24):13291. doi: 10.3390/ijms252413291 (PMC11675837; doi:10.3390/ijms252413291)
Supplement: Supplementary file 1 [file ijms-25-13291-s001.zip › ijms-3290707-supplementary.pdf]

Supplemental Table S1. The docking scores between PLE and avenanthramide derivatives [a]

Rank	Avenanthramide A		Avenanthramide B		Avenanthramide C	
	Score (kcal/mol)	RMSD[b] (Å)	Score (kcal/mol)	RMSD[b] (Å)	Score (kcal/mol)	RMSD[b] (Å)
1	-5.9	**-**	**-6.2**	**-**	-6.1	-
2	-5.8	2.055	-5.1	1.473	**-5.9**	**1.499**
3	**-5.4**	**1.426**	-3.9	1.787	-4.1	2.984
4	-5.1	1.803	-	-	-	-
5	-4.1	1.959	-	-	-	-
6	-3.2	2.497	-	-	-	-

[a]The selected binding pose was described in Figure 1. Avn C is the lowest inhibition of PLE in the experiment, we selected 1st binding pose in avenanthramide A and B, and the 3rd binding pose in avenanthramide C.

[b]RMSD (Root mean squared distance) was compared to heavy atoms of the best binding pose.

Supplemental Table S2. The total interaction energy between PLE and avenanthramide derivatives using FMO calculation

Index	Total interaction energy (kcal/mol)[a]		
	Avenanthramide A	Avenanthramide B	Avenanthramide C
1	-64.254	**-52.369**	-49.614
2	-56.877	-38.689	**-60.539**
3	**-67.209**	-4.528	62.601
4	-38.300	-	-
5	-40.223	-	-
6	0.044	-	-

[a]The bold total interaction energy was described as the minimum total interaction energy among the binding poses of each avenanthramide derivative.

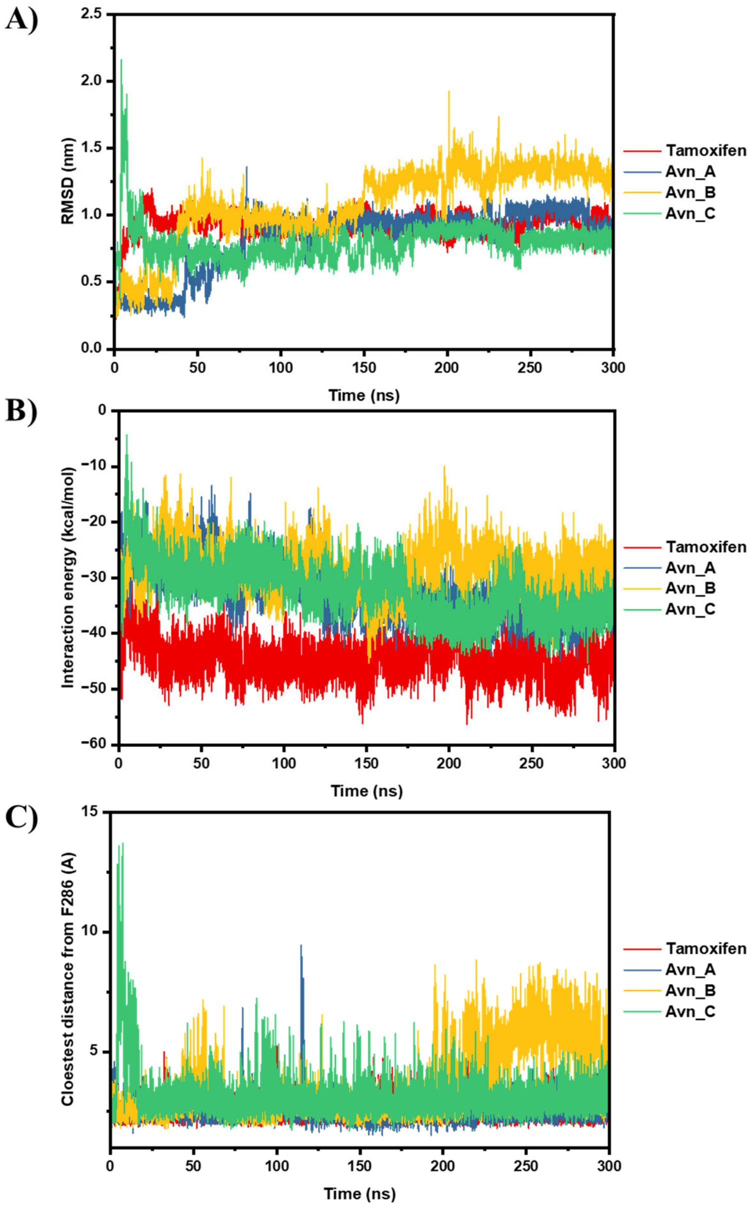

Supplemental Figure S1. Molecular dynamics simulation using PLE complex bound to tamoxifen and three avenanthramide derivative. (A) Root mean squared distance (RMSD) for each ligand. The standard deviations of RMSD by 300ns MD simulation were 0.956 Å (Tamoxifen), 2.357 Å (Avenanthramide A), 2.863 Å (Avenanthramide B), and 1.305 Å (Avenanthramide C). (B) Interaction energy between PLE and ligand calculated by gmx_MMPBSA. Average interaction energy for each ligand are -44.553 ± 3.444 kcal/mol (Tamoxifen), -32.477 ± 4.560 kcal/mol (Avenanthramide A), -28.367 ± 4.125 kcal/mol (Avenanthramide B), and -32.409 ± 4.922 kcal/mol (Avenanthramide C). (C) Closest distance between PLE and each ligand. Average closest distances are 0.253 ± 0.038nm (Tamoxifen), 0.260 ± 0.045nm (Avenanthramide A), 0.367 ± 0.152nm (Avenanthramide B), and 0.311 ± 0.095nm (Avenanthramide C). Tamoxifen and three avenanthramide derivatives are well bound to outside of active site in PLE.
